# Analysis of potential categories of sleep problems in non-dialysis patients with chronic kidney disease

**DOI:** 10.3389/fphys.2026.1744485

**Published:** 2026-04-22

**Authors:** Jie Zhou, Xiaoke Zhu, Jiamei Xu, Chunxiang Huang, Dan Liu

**Affiliations:** Department of Nephrology, Hangzhou TCM Hospital Affiliated to Zhejiang Chinese Medical University, Zhejiang, Hangzhou, China

**Keywords:** chronic kidney disease, latent class analysis, non-dialysis, phenotype-specific interventions, sleep problems

## Abstract

**Introduction:**

Sleep disturbances are highly prevalent among patients with non-dialysis chronic kidney disease (CKD) and are associated with adverse clinical outcomes. However, current research predominantly relies on aggregate scale scores, which may overlook the heterogeneity of sleep symptoms. This study aims to identify distinct latent categories of sleep problems and their influencing factors among patients with non-dialysis CKD using latent class analysis, thereby providing an evidence base for phenotype-specific interventions.

**Methods:**

From June to July 2023, a convenience sampling was used to select 405 patients from the Nephrology Department of a tertiary hospital in Hangzhou. Data were collected using a general information questionnaire, the Pittsburgh Sleep Quality Index (PSQI), the Hospital Anxiety and Depression Scale (HADS), the International Restless Legs Syndrome Assessment Scale (IRLS), and a Visual Analog Scale (VAS). Mplus 8.0 was used for latent class analysis, and unordered multinomial logistic regression analysis was performed to evaluate factors associated with the different latent classes.

**Results:**

Three latent classes were identified: “inefficient sleep and short sleep duration” (34.6%), “good sleep” (50.4%), and “low sleep quality with long sleep duration” (15.0%). Multinomial logistic regression analysis revealed that, compared with the good sleep group, age ≥45 years, skin pruritus, edema, early CKD stage, glucocorticoid or hypnotic use, anxiety and depression, and a history of COVID-19 infection were significant factors associated with sleep problem classification (*P* < 0.05).

**Conclusion:**

Sleep problems among patients with non-dialysis CKD are heterogeneous. Targeted, class-specific interventions should be developed to improve sleep quality for different patient subgroups.

## 1 Introduction

Chronic kidney disease (CKD) has emerged as a critical global public health challenge. According to the 2023 Global Burden of Disease study, CKD affects approximately 788 million adults worldwide, with an age-standardized prevalence of 14.2%, making it the ninth leading cause of death globally (Collaborators GBDCKD, 2025). In China, the burden is similarly substantial yet distinct; recent national surveillance data estimate the prevalence of CKD, kidney function impairment, and albuminuria at 8.2%, 2.2%, and 6.7%, respectively. Among Chinese adults with CKD, the disease stage distribution is skewed towards early stages, with 73.3%, 25.0%, and 1.8% categorized into stages 1-2, 3, and 4-5, respectively (Wang et al., 2023) reflecting an overall upward trend in disease burden (Wang et al., 2023).

The prolonged course of CKD precipitates multiple complications that severely impair quality of life, with sleep disturbances being among the most prevalent and debilitating. The relationship between sleep disorders and CKD is bidirectional: sleep disturbances can accelerate CKD progression, while uremic toxins and metabolic disruptions in CKD further fragment sleep architecture, creating a vicious cycle (Hemati et al., 2023; Koh et al., 2024) that exacerbates psychological distress and cardiovascular risk. However, current research on sleep problems in CKD predominantly relies on aggregate scores from standard assessment scales to define insomnia severity. This variable-centered approach often masks the internal heterogeneity of sleep symptoms, failing to distinguish distinct clinical phenotypes that may require different management strategies.

To address this limitation, Latent Class Analysis (LCA) offers a robust person-centered alternative to traditional variable-centered methods. LCA identifies unobserved subgroups based on individuals’ distinct response patterns, segmenting the population into mutually exclusive latent classes wherein members share similar profiles while remaining maximally distinct from those in other classes (Lanza et al., 2012; Sorgente et al., 2025). This methodology has gained traction in sleep research for addressing phenotypic heterogeneity, with recent studies employing LCA to characterize distinct sleep disturbance subtypes across various populations (Li et al., 2025; Tan et al., 2025).

Accordingly, this study applies LCA to identify distinct sleep quality phenotypes among non-dialysis CKD patients at our center and to elucidate factors influencing class membership. The study is guided by the “biopsychosocial model” (Engel, 1977; Bolton, 2023), which posits that health outcomes—including sleep quality—are shaped by the dynamic interplay of biological, psychological, and social factors. This framework has been widely applied in sleep research to understand the multidimensional nature of sleep disturbances (Becker et al., 2015).Based on this framework, we hypothesize that: 1) patients with non-dialysis CKD exhibit heterogeneous sleep patterns that can be classified into distinct phenotypes; 2) these phenotypes are differentially associated with biological factors (e.g., CKD stage, uremic toxins, RLS), psychological factors (e.g., anxiety, depression, COVID-19 stress), and social factors (e.g., education, occupation); and 3) understanding these phenotype-specific factors can inform targeted clinical interventions. By transcending conventional scale-based assessments that treat sleep problems as a unidimensional construct, these findings aim to provide a nuanced evidence base for early identification, phenotype-specific interventions, and personalized long-term management of sleep problems in this vulnerable population.

## 2 Methods

### 2.1 Participants

Convenience sampling was used to recruit inpatients from the Nephrology Department of Hangzhou Hospital of Traditional Chinese Medicine between June and July 2023 as study subjects. Inclusion criteria were as follows: 1) Meeting the diagnostic criteria for CKD according to the American Kidney Fund’s Clinical Practice Guidelines for Kidney Disease/Dialysis (National Kidney F, 2012) (K/DOQI) diagnostic criteria for CKD; 2) non-dialysis hospitalized patients with CKD stages 1–5 who were in stable condition; 3) participants who provided informed consent, voluntarily participated, and were able to independently complete the questionnaire; 4) age ≥ 18 years. Exclusion criteria were as follows: 1) Acute renal failure; 2) history of diagnosed psychiatric disorders; 3) communication barriers; 4) current participation in another clinical trial.

### 2.2 Sample size estimation

Based on methodological guidelines by Nylund (Nylund et al., 2007), a sample size of N ≥ 300 is generally sufficient to accurately recover latent class structures with 3–4 classes, provided the classes are well-separated and indicator quality is adequate.In this study, we anticipated identifying 3 to 4 distinct sleep quality phenotypes based on preliminary clinical observations and literature. Assuming a conservative scenario where the smallest class represents 10% of the population, a minimum of 250–300 participants would be required to secure ≥25–30 individuals per class. Furthermore, considering a potential attrition rate of 10% due to incomplete responses or exclusion criteria, the target sample size was set at 350 participants. The final analytic sample of 405 valid cases exceeds this requirement, providing adequate statistical power (>0.80) to detect distinct latent classes and ensuring the robustness of the model fit indices (AIC, BIC, aBIC) and classification accuracy (entropy).

### 2.3 Study design

This cross-sectional study employed a face-to-face survey administered via the QuestionnaireStar electronic platform on tablets. Two trained researchers collected data in strict accordance with predefined inclusion and exclusion criteria. Eligible patients received a detailed explanation of the study’s purpose, methodology, and confidentiality protocols before providing written informed consent. The survey included standardized instructions and required approximately 20 minutes to complete. A total of 407 questionnaires were distributed, yielding 405 valid responses for a response rate of 99.5%. Ethical approval was obtained from the Medical Ethics Committee of Hangzhou Hospital of Traditional Chinese Medicine (Approval No.: 202126118024002).

### 2.4 Data collection

#### 2.4.1 General information questionnaire

The questionnaire was designed by the researchers and included patients’ age, gender, educational level, marital status, occupation, primary disease, comorbidities, CKD stage, medication use, admission blood pressure, edema severity, duration of kidney disease, daytime activity level, COVID-19 infection status, nocturia, laboratory indicators (Ca, P, Hb, Pro, SCr, and Glomerular Filtration Rate), poor sleep habits, physical pain, and sensory abnormalities.

#### 2.4.2 Pittsburgh sleep quality index

PSQI is a widely used self-report instrument for assessing sleep quality, demonstrating high reliability and validity. It was developed by Buyss et al. (1989) (Buysse et al., 1989) to evaluate participants’ sleep quality during the preceding month. PSQI includes 19 items analyzed across seven components: subjective sleep quality (A), sleep latency (B), sleep duration (C), sleep efficiency (D), sleep disturbances (E), hypnotic medication use (F), and daytime dysfunction (G). Each component is scored from 0 to 3 points, and the sum of the seven components yields a total PSQI, ranging from 0 to 21 points. Higher scores indicate poorer sleep quality. In this study, the Cronbach’s alpha coefficient for PSQI was 0.844.

#### 2.4.3 Hospital anxiety and depression scale

HADS was developed by Zigmond and Snaith RP in 1983 (Zigmond and Snaith, 1983), and is primarily used to screen for anxiety and depressive symptoms among patients in general hospitals (Yamamoto-Furusho et al., 2018). It has since been widely applied across various clinical conditions (Sun et al., 2017; Annunziata et al., 2020; Lodhi et al., 2020). In this study, participants’ anxiety and depression were assessed using the Chinese-Cantonese version of HADS, which has been validated by Leung et al. (1999) against the Hamilton Rating Scale for Depression. The scale includes 14 items, with a total score ranging from 0 to 7, indicating no anxiety or depression, 8-10, indicating possible anxiety or depression, and 11-20, indicating significant anxiety or depression. In this study, the Cronbach’s alpha coefficient for HADS was 0.850.

#### 2.4.4 International restless legs syndrome rating scale

IRLS (Allen et al., 2014) assesses the severity and frequency of restless legs syndrome (RLS) symptoms, sleep disturbance, daytime sleepiness, and impact on daily activities and mood. Higher scores indicate greater symptom severity. The scale comprises 10 questions, each scored 0–4 points. Symptom severity is categorized into five levels: 0 indicates no symptoms; 1–10 is mild; 11–20 is moderate; 21–30 is severe; 31–40 is very severe. In this study, the Cronbach’s alpha coefficient for IRLS scale was 0.913.

#### 2.4.5 Visual analog scale for itching

VAS assesses the severity of skin itching in patients with non-dialysis CKD. The total score ranges from 0 to 10, with patients rating the intensity of itching during the previous week using a scale marked with 0–10 points. Scores are interpreted as follows: 0 points: No itching; 1–2 points: Mild, tolerable; 3–6 points: Moderate itching affecting sleep but remains tolerable; 7–10 points: Severe itching with intense discomfort that affects appetite and sleep (Xie et al., 2010).

### 2.5 Quality control

Rigorous quality control measures were implemented across three stages.

Pre-collection: Two researchers received standardized training on CKD and sleep assessment, including the administration of PSQI, HADS, IRLS, and VAS scales, to ensure consistent participant screening and data collection procedures.

Collection: Surveys were conducted in private settings within the nephrology ward to minimize distractions. Researchers monitored the process in real time and immediately reviewed each completed questionnaire upon submission. Participants were prompted on the spot to address any missing items, unclear responses, or logical inconsistencies (e.g., contradictory answers between sleep duration and bedtime). This face-to-face administration with immediate verification contributed to a 99.5% valid response rate (405 valid questionnaires from 407 distributed).

Data entry and cleaning: All data were double-entered independently by two research staff using EpiData software. The two datasets were compared, and any discrepancies were resolved by referring to the original paper questionnaires. Data cleaning included screening for outliers (defined as values beyond ±3 standard deviations from the mean), identifying logical inconsistencies (e.g., reported sleep duration exceeding time in bed), and excluding cases with >20% missing data on key measures (n=2). All statistical analyses, including latent class analysis, were supervised by a senior statistician to ensure methodological robustness.

### 2.6 Statistical analysis

Statistical analysis was performed using *Mplus* statistical modeling software (version 8.3) and the Statistical Package for the Social Sciences (SPSS, version 27.0). Data preprocessing and recoding were conducted using SPSS 27.0. The seven components of PSQI scale were targeted as categorical variables. Based on the 4-point Likert scale, raw item scores were converted into dichotomous variables for LCA: scores ≤ 1 were assigned to the low-response group (coded as 0); scores > 1 were assigned to the high-response group (coded as 1).

Starting from a one-class initial model, the number of latent classes was progressively increased to identify the optimal model. Model fit was evaluated using the Akaike Information Criterion (AIC), Bayesian Information Criterion (BIC), and adjusted Bayesian Information Criterion (aBIC), with lower values indicating better model fit. Entropy values (0-1) were used to assess classification precision, with values closer to 1 representing higher accuracy; an entropy value of approximately 0.8 corresponds to a classification accuracy exceeding 90%. Likelihood ratio tests, including the Log-Likelihood Ratio Test (LRT) and Bootstrap-based Likelihood Ratio Test (BLRT), were used to compare model fit differences among latent classes. A *P* < 0.05 indicated that a k-category model fit significantly better than the k-1 category model (Sinha et al., 2021; Wang et al., 2025). Based on the optimal model, multivariate logistic regression analysis was performed in SPSS to explore factors affecting latent sleep-problem classes among patients with non-dialysis CKD, stages 1-5, as the dependent variable.

## 3 Results

### 3.1 Baseline characteristics of the study population

Basic Characteristics of Participants with Anxiety, Depression, Skin Itching, and Restless Legs Syndrome: A total of 405 patients were included in this study, comprising 250 males (61.7%) and 155 females (38.3%), with a mean age of 58.7 ± 14.1 years. The primary underlying diseases were chronic glomerulonephritis (361 patients; 89.1%), diabetic nephropathy (29 patients; 7.2%), and other conditions (15 patients; 3.7%). Anxiety and depression were identified in 50 patients (12.3%), pruritus in 48 patients (11.9%), and RLS in 18 patients (4.4%).

### 3.2 Latent class analysis results and naming of latent categories of sleep problems in patients with CKD

Latent class analysis (LCA) was performed by sequentially estimating models with one to six classes. Model fit indices are summarized in **Table 1**. The Bayesian Information Criterion (BIC) decreased from the 1-class to the 3-class solution, reaching its minimum value at the 3-class model (BIC = 2619.458), and subsequently increased for the 4-, 5-, and 6-class solutions. This trend indicates that the 3-class model offers the optimal balance between model fit and parsimony.

**Table 1:** Model fitting results for latent category analysis of insomnia characteristics in patients with non-dialysis CKD (n = 405).

Model	Log (L)	AIC	BIC	aBIC	Entropy	LMR P-value	BLRT P-value	Category probability
1	-1609.716	3233.432	3261.459	3239.248	/	/	/	1.00
2	-1284.100	2598.200	2658.258	2610.661	0.874	<0.001	<0.001	0.44/0.56
3	-1240.684	2527.369	2619.458	2546.476	0.931	<0.001	<0.001	0.35/0.49/0.16
4	-1221.353	2504.706	2628.827	2530.460	0.858	0.0163	<0.001	0.49/0.19/0.18/0.14
5 6	-1215.032 -1209.858	2508.064 2513.716	2664.216 2701.898	2540.464 2552.761	0.860 0.863	0.2667 0.176	0.3333 0.6667	0.12/0.49/0.16/0.18/0.05 0.06/0.17/0.48/0.11/0.12/0.07

AIC, Akaike Information Criterion; BIC, Bayesian Information Criterion; aBIC, Adjusted Bayesian Information Criterion; Entropy, Information Entropy Index; LMRT, Log-Likelihood Ratio Test with Ruben Correction; BLRT, Bootstrap-Based Likelihood Ratio Test.

Results from the likelihood ratio tests further supported this selection. The Lo-Mendell-Rubin (LMR) and Bootstrap Likelihood Ratio Test (BLRT) yielded significant *p*-values (*p*<0.05) for the 2-class and 3-class models, indicating significant improvements in fit over the preceding models. For the 4-class solution, although the LMR test remained statistically significant (*p*=0.016), the *p*-value was notably larger than that of the 3-class comparison, suggesting a diminishing return in model improvement. Crucially, the LMR tests for the 5-class and 6-class solutions were non-significant (*p*>0.05), confirming that adding more than four classes did not provide a statistically better fit.

**Figure 1:**
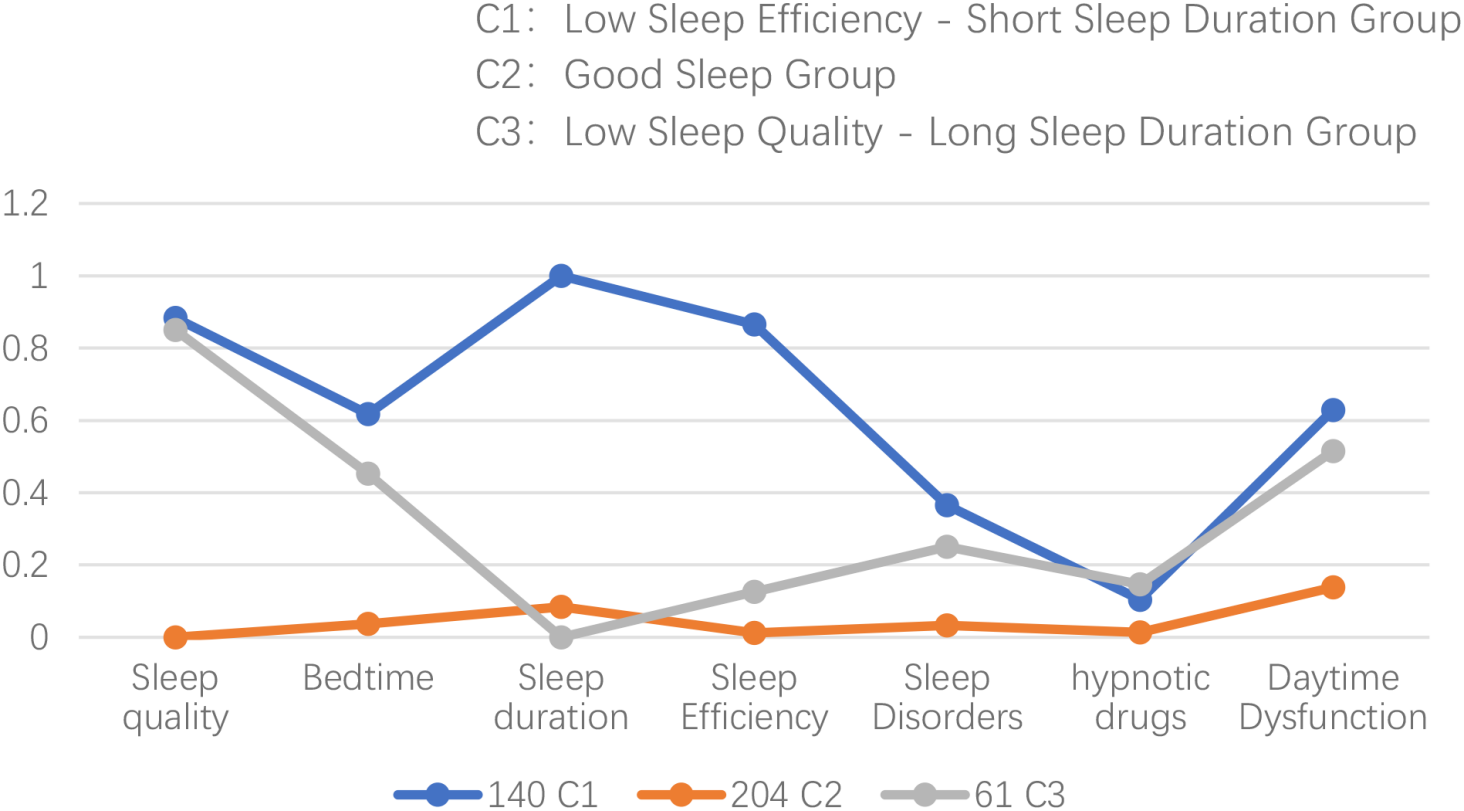
Classification analysis of sleep patterns in chronic kidney disease. PSQI scores were reverse-coded for analysis; therefore, higher values on the y-axis indicate poorer sleep quality and more severe disturbances.

Considering the minimal BIC, the significant improvement up to three classes, and the lack of substantial benefit beyond four classes, the 3-class model was determined to be optimal. This model also demonstrated excellent classification accuracy, with an entropy of 0.931 and average posterior probabilities ranging from 96.6% to 98.9% (**Table 2**). Thus, the 3-class solution was retained for subsequent analyses. The conditional response probabilities for the final model are presented in **Figure 1**.

**Table 2:** Average membership probability for each latent category (column).

Category	C1	C2	C3
C1	0.989	0.011	<0.001
C2	0.003	0.966	0.031
C3	<0.001	0.023	0.977

Diagonal values (bold) represent the average probability of correct class assignment. All values exceed 0.96, indicating excellent classification accuracy and clear separation among the three latent classes.

Class 1 (C1): This class included 140 participants, with relatively high scores across all items. Sleep efficiency and sleep duration scores were significantly higher than those of the other two groups; therefore, this class was labeled the “Low Sleep Efficiency-Short Sleep Duration Group.”; Class (C2): This class had 204 participants with relatively low scores across all items, and was labeled the “Good Sleep Group “. Class 3 (C3): This class included 61 participants, who scored higher on subjective sleep quality but lowest on sleep duration, suggesting longer sleep duration but poorer sleep quality; hence, it was labeled the “Low Sleep Quality-Long Sleep Duration Group”.

### 3.3 Univariate analysis of potential categories of sleep problems in patients with CKD with different characteristics

Univariate analysis revealed statistically significant associations (*P* < 0.05) of sleep problems with the following factors: age, education level, occupation, primary disease, comorbidities, CKD stage, edema, daytime activity level, medication use (glucocorticoids and hypnotics), history of COVID-19 infection, nocturia, physical discomfort (pain or leg twitching), anxiety/depression (HADS score), RLS, sensory abnormalities (cold/heat), itching scores, PSQI scores, serum phosphorus, hemoglobin, serum creatinine levels, and laboratory indicators related to glomerular filtration rate were statistically significant (*P* < 0.05). Other univariate factors, including gender, marital status, nocturnal breathing issues, and poor sleep habits, indicated no statistically significant differences (*P* > 0.05) (**Table 3**).

**Table 3:** Univariate analysis of general characteristics of study participants and three potential categories of sleep problems in patients with CKD.

Item	Low sleep efficiency-short sleep duration group (C1 = 140)	Good sleep group (C2 = 204)	Low sleep quality-long sleep duration group (C3 = 61)	Test statistic	*P*-value
Age,years					
<45 ≥45	9(6.4) 131(93.6)	60(29.4) 144 (70.6)	16 (26.2) 45(73.8)	*χ*^2^ = 8.623	< 0.001
Sex, n(%)					
Male	81 (57.9%)	131 (64.2%)	38 (62.3%)	*χ*^2^ = 1.431	0.489
Female	59 (42.1%)	73 (35.8%)	23 (37.7%)		
Education Level					
Junior high school or below	102 (72.9%)	112 (54.9%)	35 (57.4%)	*χ*^2^ = 23.249	0.026
High school or above	38 (27.1%)	92 (45.1%)	26 (42.6%)		
Occupation,n(%)					
Employed	72 (51.40%)	149 (73.0%)	44 (72.10%)	*χ*^2^ = 18.567	<0.001
Retired/Unemployed	68 (48.60%)	55 (27.0%)	17 (27.90%)		
Marital Status, n(%)					
Married	120 (85.7%)	176 (86.3%)	56 (91.8%)	*χ*^2^ = 11.835	0.066
Unmarried/Divorced/Widowed	20 (14.3%)	28 (13.7%)	5 (8.2%)		
Primary Disease, n(%)					
Chronic-glomerulonephritis	118 (84.3%)	180 (88.2%)	51 (83.6%)	*χ*^2^ = 29.900	<0.001
Others	22 (15.7%)	24 (11.8%)	10 (16.4%)		
Comorbidities, n(%)					
No	2 (1.4%)	19 (9.3%)	3 (4.9%)	*χ*^2^ = 9.391	0.009
Yes	138 (98.6%)	185 (90.7%)	58 (95.1%)		
CKD Stage, n(%)					
Stages 1-2	24 (17.1%)	85 (41.6%)	18 (29.5%)	*χ*^2^ = 30.453	<0.001
Stages 3-5	116 (82.9%)	119 (58.4%)	43 (70.5%)		
Edema, n(%)					
No	69 (49.3%)	140 (68.6%)	36 (59.0%)	*χ*^2^ = 15.735	0.015
Yes	71 (50.7%)	64 (31.4%)	25 (41.0%)		
Daytime Activity, n(%)					
Work	25 (17.9%)	7 (3.4%)	6 (9.8%)	*χ*^2^ = 42.428	<0.001
Household chores	44 (31.4%)	127 (62.3%)	37 (60.7%)		
Prolonged sitting or lying down	71 (50.7%)	70 (34.3%)	18 (29.5%)		
Glucocorticoid use, n(%)					
No	113 (80.7%)	178 (87.3%)	45 (73.8%)	*χ*^2^ = 6.806	0.033
Yes	27 (19.3%)	26 (12.7%)	16 (26.2%)		
Hypnotic Use, n(%)					
No	129 (92.1%)	202 (99.0%)	49 (80.3%)	*χ*^2^ = 29.349	<0.001
Yes	11 (7.9%)	2 (1.0%)	12 (19.7%)		
COVID-19 infection History, n(%)					
No	123 (87.9%)	202 (99.0%)	54 (88.5%)	*χ*^2^ = 20.275	<0.001
Yes	17 (12.1%)	2 (1.0%)	7 (11.5%)		
Poor sleep habits, n(%)					
No	123 (87.9%)	155 (76.0%)	50 (82.0%)	*χ*^2^ = 7.650	0.22
Yes	17 (12.1%)	49 (24.0%)	11 (18.0%)		
Nocturia, n(%)					
No	23 (16.4%)	59 (28.9%)	11 (18.0%)	*χ*^2^ = 8.312	0.016
Yes	117 (83.6%)	145 (71.1%)	50 (82.0%)		
Pain, n(%)					
No	108 (77.1%)	186 (91.2%)	52 (85.2%)	*χ*^2^ = 13.140	<0.001
Yes	32 (22.9%)	18 (8.8%)	9 (14.8%)		
Leg Twitching, n(%)					
No	83 (59.3%)	157 (77.0%)	46 (75.4%)	*χ*^2^ = 13.295	<0.001
Yes	57 (40.7%)	47 (23.0%)	15 (24.6%)		
Nocturnal Breathing Problems, n(%)					
No	98 (70.0%)	153 (75.0%)	40 (65.6%)	*χ*^2^ = 2.426	0.297
Yes	42 (30.0%)	51 (25.0%)	21 (34.4%)		
RLS, n(%)					
No	128 (91.4%)	201 (98.5%)	58 (95.1%)	*χ*^2^ = 9.895	0.007
Yes	12 (8.6%)	3 (1.5%)	3 (4.9%)		
Night sweats, n(%)					
No	138 (98.6%)	203 (99.5%)	61 (100.0%)	*χ*^2^ = 1.530	0.465
Yes	2 (1.4%)	1 (0.5%)	0		
Feeling cold, n(%)					
No	116 (82.9%)	189 (92.6%)	53 (86.9%)	*χ*^2^ = 7.916	0.019
Yes	24 (17.1%)	15 (7.4%)	8 (13.1%)		
Feeling hot, n(%)					
No	125 (89.3%)	198 (97.1%)	55 (90.2%)	*χ*^2^ = 9.221	0.01
Yes	15 (10.7%)	6 (2.9%)	6 (9.8%)		
Systolic BP, mmHg	137.0 ± 20.7	133.0 ± 18.0	130.9 ± 19.8	*F* = 2.754	0.065
Diastolic BP, mmHg	78.7 ± 13.6	81.4 ± 12.3	79.9 ± 13.8	*F* = 1.892	0.152
Duration of nephropathy,months	60 (1,480)	36 (1,600)	60 (1,360)	*H* = 3.273	0.195
Itch Score	0 (0, 8)	0 (0, 5)	0 (0, 8)	*H* = 17.537	<0.0001
PSQI Score	12.5 ± 3.2	3.6 ± 1.9	8.0 ± 2.5	*F* = 537.270	<0.001
Anxiety/Depression (HADS)	0 (0, 16)	0 (0, 8)	0 (0, 18)	*H* = 34.454	<0.001
Serum Phosphorus,mmol/L	1.2 ± 0.35	1.1 ± 0.28	1.15 ± 0.29	*F* = 3.984	0.019
Serum uric acid,μmol/L	397.3 ± 115.6	404.6 ± 119.1	392.1 ± 115.0	*F* = 0.328	0.721
Hemoglobin,g/L	107.4 ± 23.1	117.7 ± 24.0	109.7 ± 23.9	*F* = 8.483	<0.001
Serum calcium,mmol/L	2.14(1.7,3.3)	2.14(1.6,2.67)	2.1 (1.76, 2.56)	*H* = 5.188	0.075
Serum creatinine,μmol/L	184.5 (45,943)	132 (97, 904)	174 (39,802)	*H* = 11.050	0.004
Urinary protein,g/24 h	1.48(0.01,27.26)	1.26(0.04,18.93)	1.4 (0.03, 12.96)	*H* = 1.965	0.374
GFR,ml/min/1.73 m^2^	25.2 (4.7, 122.5)	46.8 (3.2, 167.3)	30.6 (4.5, 150)	*H* = 14.598	0.001

Data are presented as n (%) for categorical variables, mean ± standard deviation (SD) for normally distributed continuous variables, or median (range) for non-normally distributed continuous variables.

CKD, chronic kidney disease; RLS, restless legs syndrome;BP, blood pressure; PSQI, Pittsburgh Sleep Quality Index; HADS, Hospital Anxiety and Depression Scale; eGFR, estimated glomerular filtration rate.

### 3.4 Multivariate logistic regression analysis of latent classes of sleep problems in patients with non-dialysis CKD

Collinearity diagnosis indicated that all tolerance values exceeded 0.1. Using the latent sleep problem classes of patients with non-dialysis CKD as the dependent variable, indicators with *P* < 0.05 from univariate analysis were included as independent variables in a multinomial unordered logistic regression. The C2 class (good sleep) was used as the reference group. The results indicate that age, stage of CKD, skin itching, use of glucocorticoids and hypnotics, history of COVID-19 infection, RLS, edema, and anxiety-depression (HADS score) were significant factors associated with the latent classes of sleep problems in patients with non-dialysis CKD (**Table 4**).

**Table 4:** Multiclass disordered logistic regression analysis of sleep problems in patients with non-dialysis CKD (n = 405).

	Low sleep efficiency–short sleep duration group vs. good sleep group						Low sleep quality–long sleep duration group vs. good sleep group	
Item	*ß*	*OR* Value	95% *CI*	*P*	*ß*	*OR* Value	95% *CI*	*P*
Age								
<45 ≥45	1.766	1.0 5.848	1.944-17.597	0.002	0.274	1.0 1.315	0.476-3.635	0.597
Skin Itching								
Yes No	1.074	2.928 1.0	1.066-8.043	0.037	0.283	1.327 1.0	0.367-4.801	0.666
CKD Stage								
Stages 1-2 Stages 3-5	-1.342	0.261 1.0	0.083-0.827	0.022	-1.789	0.167 1.0	0.039 -0.717	0.016
Glucocortic-oid Use								
Yes No	1.128	3.091 1.0	1.380-6.924	0.006	2.516	2.516 1.0	1.009-6.275	0.048
Anxiety/Depressi-on (HADS)								
Yes No	3.158	23.521 1.0	7.630-72.505	<0.001	2.630	13.878 1.0	4.202-45.832	<0.001
Edema								
Yes No	0.858	2.359 1.0	1.320-4.213	0.004	0.593	1.809 1.0	0.885-3.699	0.104
COVID-19 infection History								
Yes No	2.793	16.331 1.0	3.245-82.183	0.001	2.806	16.5401.0	2.998-91.256	0.001
RLS								
Yes No	2.547	12.775 1.0	1.427-114.395	0.023	1.992	7.332 1.0	0.755-71.159	0.086
Hypnotic Use								
Yes No	1.405	4.078 1.0	0.799-20.811	0.091	2.994	19.956 1.0	3.821-104.218	<0.001

## 4 Discussion

### 4.1 Principal findings and theoretical framework

This study identified three distinct sleep phenotypes among patients with non-dialysis CKD using latent class analysis: Class 1, “Low Sleep Efficiency-Short Sleep Duration” (34.6%); Class 2, “Good Sleep” (50.4%); and Class 3, “Low Sleep Quality-Long Sleep Duration” (15.0%). Multivariate analysis revealed distinct factor profiles. Class 1 was uniquely characterized by older age, skin itching, edema, and restless legs syndrome (RLS). Class 3 was uniquely characterized by hypnotic use. Both adverse phenotypes shared advanced CKD stage, glucocorticoid use, anxiety/depression, and history of COVID-19 infection—though the association with anxiety/depression was substantially stronger in Class 1 (OR 23.521) than in Class 3 (OR 13.878).

**Figure 2:**
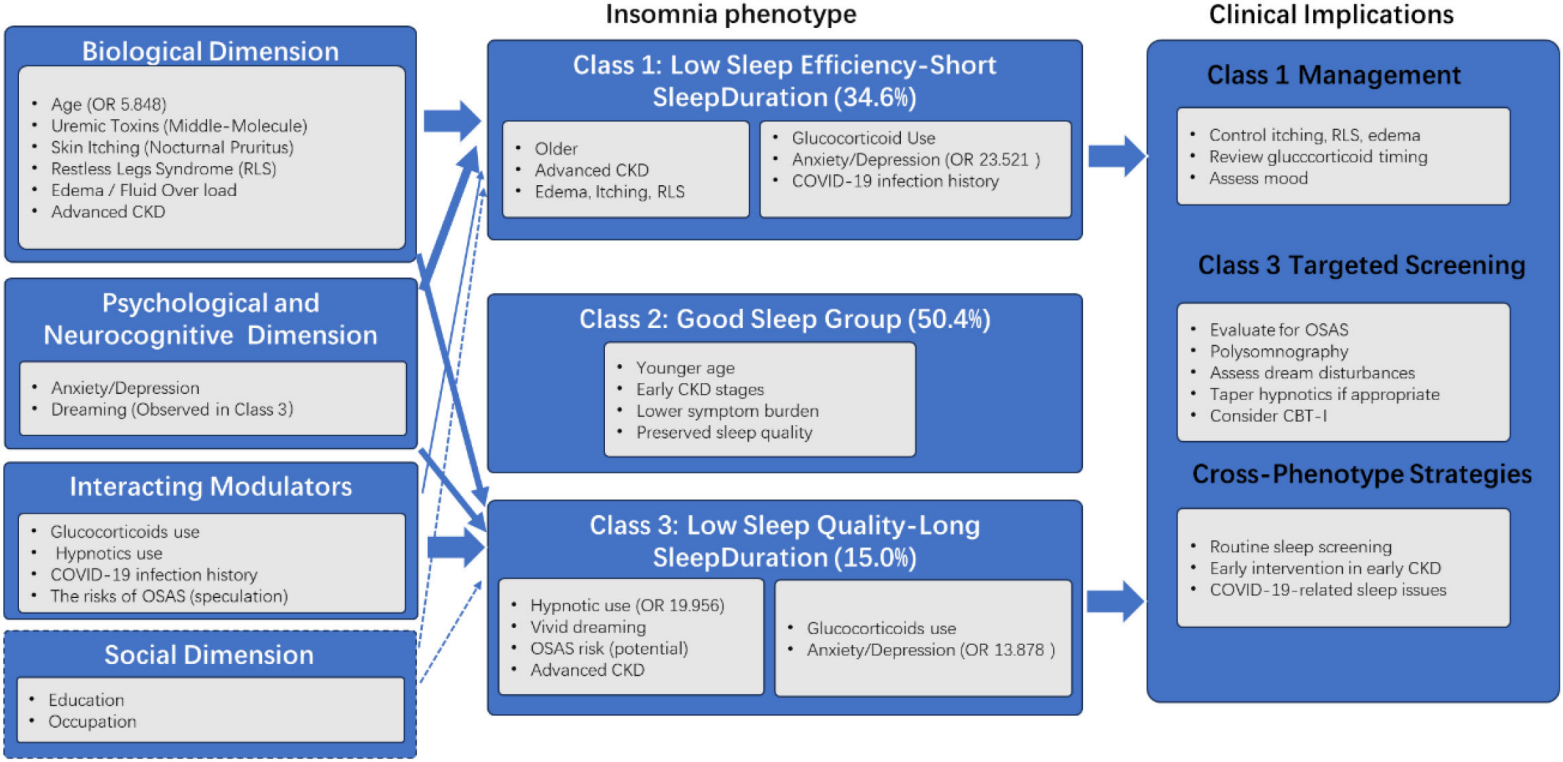
Biopsychosocial model of sleep phenotypes in patients with non-dialysis CKD. The model illustrates the multidimensional factors influencing the three identified sleep phenotypes. The biological dimension, psychological dimension, and social dimension interact to shape Class 1 (Low Sleep Efficiency-Short Sleep Duration), Class 2 (Good Sleep), and Class 3 (Low Sleep Quality-Long Sleep Duration). Arrow thickness reflects the relative strength of association based on multivariate analysis, the dotted line indicates a potential impact. Clinical intervention strategies for each phenotype are shown on the right. OR, odds ratio; CKD, chronic kidney disease; RLS, restless legs syndrome; OSAS, obstructive sleep apnea syndrome.

Viewed through the biopsychosocial model (Elder et al., 2023; Kallem et al., 2025), these phenotypes reflect the complex interplay of biological vulnerability, psychological distress, and external modulators. The conceptual framework guiding this interpretation is illustrated in **Figure 2**. Class 1 appears predominantly driven by physiological burden with reactive psychological distress, whereas Class 3 represents a convergence of medication effects, neurocognitive disturbances, and undiagnosed sleep pathology. Social factors such as education and occupation were significant in univariate analysis but not retained in the final model, suggesting their influence may operate indirectly through biological or psychological pathways.

### 4.2 Biological determinants: symptom burden and disease progression

Class 1 was uniquely characterized by older age, skin itching, edema, and RLS—a cluster of somatic symptoms pointing to a shared pathophysiology rooted in uremic toxicity.

Age-related changes in sleep architecture. Older age was a strong predictor for Class 1 (OR 5.848). Advancing age is associated with reduced slow-wave sleep, increased nocturnal awakenings, and attenuated circadian rhythms (Bai et al., 2025), which may amplify uremic toxin effects and render older patients particularly susceptible to sleep disruption (Yoshimoto et al., 2021; Guo et al., 2022). Notably, most patients in this cohort developed CKD and insomnia during adulthood; only one patient had carried the disease since childhood. Thus, the observed age effects primarily reflect aging-related vulnerability rather than cumulative disease duration. Whether pediatric-onset CKD leads to distinct sleep trajectories—shaped by prolonged toxin exposure, chronic inflammation, and the psychological demands of growing up with illness—remains an open question warranting further investigation (Craig et al., 2022).

Uremic toxins and peripheral symptoms. As CKD advances, accumulation of uremic toxins—particularly middle−molecule toxins such as β2−microglobulin and protein−bound solutes—can directly disrupt neuronal excitability and circadian regulation (Natale et al., 2021; Yamamoto et al., 2024). These toxins also drive peripheral symptoms including pruritus and RLS, which provoke frequent nocturnal awakenings (P et al., 2021; Zhang et al., 2023; Gela et al., 2024; Nalankilli et al., 2025). Pruritus severity correlates with toxin levels, and its characteristic nocturnal exacerbation disrupts sleep when rest is most needed (Zhang et al., 2023; Yamamoto et al., 2024). Pediatric nephrology has long emphasized that strategies to clear these toxins should begin before dialysis starts (Craig et al., 2022); for adults with non-dialysis CKD, optimizing enteric toxin clearance—through dietary interventions or emerging therapies—may offer a path to quieter nights, though this remains to be tested (Sumida et al., 2022; Wakino et al., 2025).

Edema and sleep disruption. Edema disrupts sleep through two interrelated mechanisms: increased nocturnal urine output (exacerbating nocturia) and, when severe, positional dyspnea that forces a semi−recumbent posture, fragmenting sleep architecture (da Silva et al., 2017; Kalantar-Zadeh et al., 2022). This fluid overload also contributes to obstructive sleep apnea pathogenesis through nocturnal rostral fluid shift—a point to which we return in the discussion of Class 3.

RLS as a treatable contributor. RLS affects 20-50% of patients with CKD and often reflects impaired iron metabolism, representing a potentially treatable contributor to sleepless nights (Elangovan et al., 2025). Its unique association with Class 1 underscores the importance of screening for iron deficiency and considering targeted interventions (P et al., 2021).

The protective effect of early CKD stage. Early CKD stage was protective against both adverse phenotypes, suggesting a critical window for intervention before biological drivers become entrenched (Vendeville et al., 2025).

### 4.3 Psychological and neurocognitive mechanisms

Class 3 presents a paradox: patients sleep the longest yet wake feeling the worst. Unlike Class 1, physical symptoms such as itching, edema, and RLS were not significant predictors. Instead, this phenotype was associated with anxiety/depression (OR 13.878) and hypnotic use (OR 19.956)—the latter addressed in the following section.

Vivid dreaming as a neurocognitive marker. During this study, we observed that patients in Class 3 frequently reported vivid, disturbing dreams that left them feeling sleep had not been restorative. We hypothesize that this reflects REM sleep dysregulation induced by uremia. Accumulating uremic toxins may disrupt cholinergic and serotonergic pathways—key modulators of REM sleep—prolonging REM density and contributing to excessive dream recall (Pilato et al., 2025; Stucky, 2025). Excessive dream recall often signals sleep fragmentation, as dreams are more likely to be remembered when individuals awaken during or immediately after REM sleep (Stucky, 2025). In CKD, such dreaming may also arise from corticosteroid use, direct neurotoxic effects of uremic toxins, or the psychological burden of chronic illness (Pilato et al., 2025). Higher symptom burden—particularly itching and RLS—may further contribute to poor sleep quality despite adequate duration, consistent with evidence that sleep duration alone does not determine self-reported sleep quality (Walton et al., 2025).

Anxiety and depression: shared but with different weights. Although anxiety and depression were significant predictors for both adverse phenotypes, the odds ratio for Class 1 (OR 23.521) was nearly twice that for Class 3 (OR 13.878). This disparity suggests that psychological distress may be more reactive in Class 1—driven by overwhelming physical discomfort—whereas in Class 3, distress may be more closely intertwined with neurocognitive alterations and medication effects (Saguban et al., 2025; Hansen et al., 2026).

Social factors as potential modulators. In univariate analysis, education and occupation were significantly associated with sleep phenotypes but were not retained in the multivariate model. This suggests their effects may operate indirectly—for example, through health literacy, medication adherence, or psychological distress—or may require larger samples to detect. Future research should incorporate comprehensive measures of socioeconomic status to better understand how social context shapes sleep in this population.

### 4.4 Interacting modulators: bridging biological and psychological domains

Beyond the core biological and psychological drivers discussed above, several external factors—glucocorticoids, hypnotics, COVID-19, and undiagnosed sleep pathology—further modulated sleep phenotypes. These factors are not confined to a single dimension of the biopsychosocial model; rather, they operate across its boundaries, illustrating the dynamic interplay between biological vulnerability, psychological stress, and medical interventions.

#### 4.4.1 Iatrogenic factors: glucocorticoids and hypnotics

Glucocorticoids were associated with both adverse phenotypes, suggesting their sleep−disrupting effects operate through shared biological pathways, including hypothalamic−pituitary−adrenal (HPA) axis activation and disruption of circadian clock gene expression (Lightman and Conway-Campbell, 2024; Seighali et al., 2024). While essential for managing many kidney diseases, their impact on sleep architecture underscores the need to balance therapeutic benefits with potential sleep−related side effects.

Hypnotic use was uniquely associated with Class 3 (OR 19.956), pointing to a group heavily reliant on sleep medications yet still reporting poor sleep quality. This iatrogenic factor operates at the intersection of biological and psychological domains. Biologically, benzodiazepines and Z−drugs suppress slow−wave sleep and alter sleep architecture, diminishing restorative function even as total sleep time increases (Koh et al., 2024; Lightman and Conway-Campbell, 2024). Psychologically, their use may reflect underlying anxiety or distress, yet chronic use can paradoxically perpetuate sleep disturbance through tolerance and dependence. Moreover, in patients with undiagnosed obstructive sleep apnea, hypnotics may be particularly problematic, as sedatives can suppress protective arousal responses to apnea (Koh et al., 2024).

#### 4.4.2 COVID-19 as a dual−hit stressor

A history of COVID-19 infection was strongly associated with both adverse phenotypes (OR ~16 for each), highlighting a mechanism that spans biological and psychological domains. Biologically, SARS−CoV−2 can invade the central nervous system, potentially disrupting the suprachiasmatic nucleus and triggering neuroinflammation that destabilizes sleep−regulatory centers (Talkington et al., 2024). Psychologically, the lived experience of COVID−19—marked by fear, isolation, and uncertainty—activates stress pathways that maintain the HPA axis in a state of sustained alert (Elder et al., 2023). For patients with CKD, whose physiological reserve may already be diminished, the added burden of COVID−related sleep disruption may be especially consequential. This “double−hit” mechanism underscores the importance of considering pandemic history in sleep assessments for this vulnerable population.

#### 4.4.3 Obstructive sleep apnea as a plausible underlying mechanism

The hallmark of Class 3—long time in bed coupled with poor subjective sleep quality—also raises the possibility of undiagnosed obstructive sleep apnea syndrome (OSAS). Although not directly measured in this study, OSAS offers a plausible biological explanation for the discrepancy between sleep duration and perceived sleep quality.

Nearly 40% of patients with CKD have OSAS, with rates rising to 47% among those aged 60 years or older (Liu et al., 2025). The mechanisms linking CKD and OSAS are multifaceted: volume overload leads to rostral fluid shift during recumbency, narrowing the upper airway; metabolic acidosis may blunt respiratory drive; and obesity and diabetes are highly prevalent (Elangovan et al., 2025). In OSAS, recurrent apneas fragment sleep architecture, leaving patients exhausted despite spending extended hours in bed. Importantly, respiratory events frequently occur during REM sleep, which may further explain the vivid dream recall observed in Class 3. Moreover, as noted above, hypnotic use in patients with undiagnosed OSAS can be particularly problematic, as sedatives may suppress protective arousal responses to apnea (Koh et al., 2024).

### 4.5 Clinical implications: a precision medicine approach

The heterogeneity revealed by this study calls for a shift from one−size−fits−all sleep management toward stratified care aligned with phenotype.

Routine screening. Clinicians should ask not only whether patients sleep poorly, but how. Simple questions—”Do you have trouble falling asleep?” “Do you feel rested after sleeping?”—can help distinguish between phenotypes.

Class 1 management. Prioritize symptom control: treat itching and RLS, manage edema (including addressing volume overload), review glucocorticoid timing to minimize nighttime disruption, and assess mood. When somatic symptoms improve, sleep often follows.

Class 3 management. Focus on medication review and evaluation for undiagnosed sleep pathology. Given the strong association with hypnotic use, consider tapering unnecessary sedatives. Screen for OSAS using tools such as STOP−Bang, and consider polysomnography when clinically indicated. Address dream disturbances, which may signal REM dysregulation or sleep fragmentation, and consider cognitive behavioral therapy for insomnia (CBT–I) when psychological factors are prominent.

COVID−19−related sleep issues. For patients with a history of COVID−19, inquire about sleep changes since infection; the virus may have left more than antibodies.

Early intervention. Early CKD stages offer a window of opportunity for protecting sleep. Intervening before biological and psychological drivers become entrenched could alter the disease trajectory for years to come.

### 4.6 Limitations

Several limitations warrant consideration. First, the cross−sectional design precludes causal inference; longitudinal studies are needed to elucidate how these phenotypes evolve over time. Second, sleep assessment relied on self−report (PSQI) rather than objective measures such as actigraphy or polysomnography, which may introduce recall bias and cannot definitively diagnose OSAS. Dream assessment was also based on patient report without standardized instruments—a reminder that this terrain remains to be mapped. Third, the single−center design and regionally specific sample may limit generalizability. Fourth, with only one patient carrying CKD since childhood, we could not examine how decades of disease shape sleep differently—an important question as young patients transition from pediatric to adult care. Finally, despite adjusting for multiple covariates, residual confounding from unmeasured factors (e.g., detailed medication history, caffeine and alcohol consumption, shift work) cannot be excluded.

## 5 Conclusion

This study identified three distinct sleep phenotypes in non-dialysis CKD patients. Class 1 reflects biological burden—advanced disease, somatic symptoms (itching, edema, RLS)—with psychological distress often reactive to physical discomfort. Class 3 represents a more complex picture: patients who sleep long yet poorly, shaped by psychological distress, neurocognitive disturbances (vivid dreaming as a marker of REM dysregulation), and iatrogenic factors (glucocorticoids, hypnotics). COVID−19 history was strongly associated with both adverse phenotypes. A stratified approach to sleep management—considering phenotype rather than merely presence of sleep problems—may enable more personalized interventions and improve outcomes in this population.

## Data Availability

The original contributions presented in the study are included in the article/supplementary material. Further inquiries can be directed to the corresponding author.
